# Vascular Contractile and Structural Properties in Diet-Induced Atherosclerosis-Prone CB_1_-LDL Receptor Double Knockout Animal Model

**DOI:** 10.3390/biomedicines14020284

**Published:** 2026-01-27

**Authors:** Kinga Shenker-Horváth, Zsolt Vass, Bálint Bányai, Stella Kiss, Kinga Bernadett Kovács, Judit Kiss, Andrea Petra Trenka, Janka Borbála Gém, Annamária Szénási, Eszter Mária Horváth, Zoltán Jakus, György L. Nádasy, Gabriella Dörnyei, Mária Szekeres

**Affiliations:** 1 Department of Morphology and Physiology, Faculty of Health Sciences, Semmelweis University, 17 Vas Street, 1088 Budapest, Hungary; shenker-horvath.kinga@tf.hu (K.S.-H.); vass.zsolt@semmelweis.hu (Z.V.); k.stella1114@gmail.com (S.K.); kiss.judit2@semmelweis.hu (J.K.); trenkandi@gmail.com (A.P.T.); poloskeine.szenasi.annamaria@semmelweis.hu (A.S.); dornyei.gabriella@semmelweis.hu (G.D.); 2 Center for Sports Nutrition Science, Hungarian University of Sports Science, 42-48 Alkotás Street, 1123 Budapest, Hungary; 3 Department of Physiology, Faculty of Medicine, Semmelweis University, 37-47 Tűzoltó Street, 1094 Budapest, Hungary; banyai.balint.peter@semmelweis.hu (B.B.); kovacs.kinga.bernadett@gmail.com (K.B.K.); gemnana707@gmail.com (J.B.G.); horvath.eszter.maria@semmelweis.hu (E.M.H.); jakus.zoltan@semmelweis.hu (Z.J.); nadasy.gyorgy.laszlo@semmelweis.hu (G.L.N.)

**Keywords:** atherosclerosis, CB_1_ receptor, LDL receptor, endocannabinoid, vascular remodeling, high-fat diet

## Abstract

**Background**: Atherosclerosis forms the background of several cardiovascular pathologies. LDL receptor knockout (LDLR-KO) mice kept on a high-fat diet (HFD) develop high cholesterol levels. Previously we found that vasodilation responses in HFD LDLR-KO mice were improved in the absence of type 1 cannabinoid receptors (CB_1_Rs). We aimed to reveal the effects of HFD and CB_1_Rs on vascular contractile and structural properties. **Methods**: Experiments were performed on LDLR-CB_1_R double knockout and wild type (WT) mice, kept on an HFD or control diet (CD) for 5 months. Thoracic aortas were isolated for Oil Red plaque staining and abdominal aorta segments for myography to obtain phenylephrine (Phe)-induced (100 nM–10 µM) contractile responses. Aorta samples were subjected to histology stainings with hematoxylin–eosin and resorcin–fuchsin (elastin density) and for smooth muscle actin (SMA) immunohistochemistry. **Results**: Phe-induced contractions significantly increased in HFD groups (*p* < 0.05) similarly in all genotypes. However, contractions were stronger with CD in CB_1_R-KO compared to WT. Plaque areas were increased in LDLR-KO mice compared to WT, significant in HFD groups (*p* < 0.05). SMA increased to HFD, while elastin density remained similar, with the highest value in double KO-HFD. Intima/media ratio significantly decreased in double KO-HFD vs. CD. **Conclusions**: Our results indicate that HFD-treated LDLR-KO mice develop atherosclerosis with functional contractile and structural alterations modulated by CB_1_Rs: absence of CB_1_Rs elicited higher contraction properties with some modification in vascular remodeling indicating contribution of the CB_1_R to cellular signalization controlling wall thickness and elasticity in pathological conditions.

## 1. Introduction

Cardiovascular diseases are leading causes of morbidity and mortality worldwide [1,2]. Among them, dyslipidemia and elevated plasma cholesterol and low-density lipoprotein (LDL) levels and consequently atherosclerosis and hypertension frequently develop which later may be associated with ischemic heart diseases and stroke. Atherosclerosis is in the background of the majority of cardiovascular and cerebrovascular diseases. It is a chronic, systemic and inflammatory disease, mainly affecting large and medium-sized arteries [3,4,5,6,7]. Atherosclerosis also may pathologically alter vascular functions by causing endothelial dysfunction [2,5,8,9,10,11,12,13,14]. Therefore, early detection, diagnosis, and treatment should be the primary focus of medical research.

To examine atherosclerosis, genetically modified experimental models of mice are widely applied [13,15,16,17]. Such models include the Apo-E-knockout mouse strain, LDL receptor-knockout (LDLR-KO) mice and Apo-E–LDLR double-KO mice. LDL-receptor-deficient (LDLR-KO) mice are frequently used in which hypercholesterolemia and atherosclerosis effectively develop. The pathology is manifested with atherosclerotic plaques deposited in the aorta if the animals are fed with high-fat, western-type diet (WTD) [13,16]. LDLR-KO mice also develop pathologic lipid profiles, decreased high-density lipoprotein (HDL) levels, and elevated very-low-density lipoprotein (VLDL) levels. LDLR-KO mice kept on a high-fat diet (HFD) are considered relevant mouse models for the human familial hypercholesterolemia and they are commonly used in animal experiments investigating hyperlipidemia and atherosclerosis. Cholesterol levels of this animal model may reach as high as 800–1000 mg/dL if mice are kept on a long-term HFD [7,13,16,17,18,19,20]. LDLR-KO mice kept on an HFD develop atherosclerotic plaques in their thoracic aorta with altered endothelium-mediated vasodilation in their arteries [12,13,16]. They also develop altered vasodilation [20].

The endocannabinoid system (ECS) is involved in the regulation of several physiological functions. It was first identified as regulator in the nervous system of synaptic transmission by retrograde action [21,22]. Since then it has been introduced that ECS is involved in other physiological mechanisms such as in the cardiovascular function, in gastrointestinal and metabolic regulation processes, food intake, involving signaling mechanisms mediated by type 1 cannabinoid receptors (CB_1_Rs) [9,23,24,25,26]. Among the endocannabinoids released from neural and vascular tissues, anandamide (arachidonoyl ethanolamide, AEA) and 2-arachidonylglycerol (2-AG) have been found to be most important. CB_1_ receptors are found in several peripheral tissues, such as the heart and the walls of blood vessels. In the ECS AEA, 2-AG and CB_1_R agonists such as tetrahydrocannabinol (THC) and synthetic agonist WIN55,212 induce vasodilator, negative inotropic and hypotensive actions via the activation of CB_1_R signaling, mostly in low dosages [9,23,24,27,28,29,30,31,32,33]. Previous studies from our and other laboratories indicate that 2-AG can be released by stimulation of calcium-generating G protein-coupled receptors (e.g., angiotensin type 1 receptors) via the activation of the enzyme diacylglycerol (DAG). The product 2-AG by acting on vascular CB_1_Rs then attenuates the agonist angiotensin II-induced vasoconstriction. At the same time it exerts a negative inotropic effect [24,25,27,34,35]. In order to investigate ECS function in atherosclerosis, we have recently established an LDLR-CB_1_R double knockout hypercholesterolemic mouse model, on which we previously found that altered vasodilator functions were effectively improved in the absence of CB_1_Rs that may suggest the beneficial action of missing receptor functions [13].

Based on our previous observation, in the present study our aim was to investigate the role of HFD and CB_1_ receptors on vascular contractile function and structural vascular remodeling, by using LDLR-CB_1_R double knockout mouse model of hypercholesterolemia-induced atherosclerosis. We hypothesized that the absence of CB_1_Rs might provide a vascular remodeling effect which can be beneficial in atherosclerosis.

## 2. Materials and Methods

### 2.1. Chemicals

Adrenergic alpha receptor agonist phenylephrine (Phe), vasorelaxant acetylcholine and other salts and chemicals were purchased from Merck KGaA (Darmstadt, Germany). Stock solutions were prepared in distilled water and subsequently diluted in Krebs solution on the day of the experiment. Krebs solution and high-potassium Krebs solution were prepared using the following components: NaCl, KCl, CaCl_2_·2H_2_O, MgSO_4_·7H_2_O, NaHCO_3_, KH_2_PO_4_, EDTA, and glucose. For a list of the immunohistological reagents and their sources and dilutions, see the corresponding subchapters. Phenylephrine was diluted in Krebs from stock solution. Krebs and K-Krebs solutions were made on the day of the experiment from the following components: NaCl, KCl, CaCl_2_·2H_2_O, MgSO_4_·7H_2_O, NaHCO_3_, KH_2_PO_4_, EDTA, and glucose. For a list and the sources of histological and immuno-histological reagents and their dilutions, see the corresponding subchapters.

### 2.2. Animals

Double knockout LDLR-KO (LDLR−/−)-CB_1_R-KO (CB_1_R−/−) mice and their wild type counterparts LDLR-WT (LDLR+/+)-CB_1_R-WT (CB_1_R+/+) were obtained by crossing heterozygous CB_1_R+/− mice with C57BL/6J background with LDLR-KO homozygous mice [13]. CB_1_R-KO mouse strain was provided by Andreas Zimmer (Cnr1tm1zim, [36]). LDLR-KO mice were obtained from Jackson Laboratory (B6.129S7-Ldlrtm1Her/J, Jackson Laboratory, Bar Harbor, ME, USA). Animals were bred at the HUN-REN Institute of Experimental Medicine’s animal house and in the animal facility of the Semmelweis University Basic Medical Science Center. Institutional and national guidelines for animal care and breeding were followed, and the study protocol was approved by the Animal Care Committee of the Semmelweis University, Budapest, and by Hungarian authorities (approval no. PE/EA/1428-7/2018 and PE/EA00670-6/2023). Thus, by crossing the LDLR-KO and CB_1_R-KO mouse strains, we established a mouse model suitable for studying the impact of CB_1_Rs in the development and atherosclerosis.

In our experiments, male mice were fed with special diets obtained from Ssniff Spezialdiäten GmbH (Soest, Germany), which were given to the animals ad libitum. Mice were genetically tested and grouped according to their genotype and diet as indicated in Table 1. Groups 1–4 were fed by a control diet (CD) containing 0% cholesterol and decreased levels of crude fat (5.1%), 11% sugar, 18.3 MJ/kg gross energy, and 15.7 MJ/kg metabolizable energy, while groups 5–8 were fed with an HFD (Western-type diet) containing elevated levels of crude fat (21.1%), 0.21% cholesterol, 34.3% sugar, 21.8 MJ/kg gross energy, and 19.1 MJ/kg metabolizable energy. Mice obtained diets from the age of 1 month until 6 months. HFD administered for this length of time has been shown to significantly increase cholesterol levels and plaque formation in LDLR-KO mice [37,38]. The termination age of the mice was uniformly 6 months.

During the experiments, mice were subjected to wire myography of their aortic rings and histochemical and immunohistochemical staining in their aortic specimens (see later).

### 2.3. Myography and Preparation

Animals were anesthetized by Euthasol, 55 mg/kg, administered intraperitoneally. The depth of anesthesia was checked by verifying the absence of pain reflexes. The whole circulatory system was perfused with Krebs to remove blood, and aortas were dissected. Thoracic aortas were removed for Oil Red plaque staining and put into 4% formaline. Upper part of abdominal aorta was removed and subjected to histochemistry and immunohistochemistry. Abdominal aortic segments below the renal arteries were removed for wire myography measurements, as described before [13,24,33,37]. Segments were put into cold Krebs solution containing the following (in mmol/L): 119 NaCl, 4.7 KCl, 2.5 CaCl_2_·2H_2_O, 1.17 MgSO_4_·7H_2_O, 20 NaHCO_3_, 1.18 KH_2_PO_4_, 0.027 EDTA, and 10.5 glucose. Abdominal aortic rings were cut and mounted onto the wires of the myograph system (610 M Multiwire Myograph System, Danish Myo Technology A/S, Aarhus, Denmark) to record isometric tension. Data were recorded simultaneously on 8 channels via the PowerLab data acquisition system; evaluations were carried out using the LabChart version 8 evaluation software (ADInstruments, Oxford, UK; Ballagi LTD., Budapest, Hungary). The myograph chambers were filled with Krebs solution and maintained at a temperature of 37 °C, aerated with carbogenic gas (95% O_2_ + 5% CO_2_) to keep the pH at 7.4. According to our protocols [13,24,33], abdominal aortic segments were pre-stretched to 10 mN and were allowed to equilibrate for 30 min. After the equilibration period, a reference contraction (considered 100%) was elicited using hyperkalemic Krebs solution, containing 124 mmol/L potassium. Phenylephrine-induced dose-dependent contractions were obtained (from 1 nmol/L to 10 μmol/L) to obtain functional contraction property. All segments were checked for functional viability (contraction and relaxation abilities).

### 2.4. Histological and Immunohistochemical Stainings

Sections of abdominal aorta, 3 μm in thickness were prepared from paraformaldehyde (PFA)-fixed, paraffin-embedded tissue. Hematoxylin–eosin staining (HE) was employed for topographical investigation, including the intima–media ratio and wall thickness. Incubation was conducted with hematoxylin (Hematoxylin modified to Gill II, Sigma-Aldrich, St. Louis, MO, USA; Eosin Y, Merck Millipore, Burlington, MA, USA). Elastic fibers were stained with resorcin–fuchsin (RF, Electron microscopy Sciences, Hatfield, PA, USA). These stainings have been used to measure the morphological parameters. Sections underwent immunohistochemistry staining for α-smooth muscle actin (SMA). Following deparaffinization, antigen retrieval was performed by heating the slides in citrate buffer (pH 6). Endogenous peroxidase activity was inhibited with 3% H_2_O_2_. To mitigate nonspecific labeling of the secondary antibody, we employed a 2.5% normal horse serum blocking solution (Vector Biolabs, Burlingame, CA, USA) for one hour. The anti α-SMA primary antibody (mouse monoclonal antibody) 1:10,000 (Abcam RRID: AB_262054, Cambridge, UK) was used with overnight application at 4 °C. On the second day for secondary labeling, we used horseradish peroxidase (HRP)-linked anti-mouse IgG polyclonal antibodies (Vector Biolabs, Burlingame, CA, USA). Visualization was conducted utilizing 3′3-diaminobenzidine (DAB, Vector Biolabs, Burlingame, CA, USA). Slides were captured using a Nikon Eclipse Ni-U microscope equipped with a DS-Ri2 camera (Nikon, Minato, Tokyo, Japan). Photographs of the slides were captured at 10× magnification in every case of the staining procedures. The thickness of the aorta wall, as well as the individual thicknesses of the intima and media layers, were measured on HE slides. Noncalibrated optical density (O.D.) was calculated on the HE, RF and SMA-stained slides. Elastin area as % of medial cross sectional area was calculated and defined as elastic density. On immunohistochemical slides, the brown positivity and background staining (DAB and hematoxylin) were separated. For all measurements on the stainings we used the FIJI® software, ImageJ 1.54f; Java 1.8.0_322, (National Institutes of Health, Bethesda, MA, USA).

### 2.5. Oil Red Staining and the Analysis of Atherosclerotic Plaques

We subjected the isolated and cleaned aortas to Oil Red staining (Merck KGaA, Darmstadt, Germany), in order to visualize atherosclerotic plaques. Aortas were fixed in 4% PFA for 24 h at 4 °C. The fixed samples were then rinsed with phosphate-buffered saline (PBS) (137 mM NaCl; 2.7 mM KCl; 10 mM Na_2_HPO_4_; and 1.8 mM KH_2_PO_4_; pH: 7.4) once. PBS was then discarded and we washed the aorta samples with 60% isopropanol for 20 s on a horizontal shaker. After removing the 60% isopropanol we added Oil Red O staining solution to the aortas and incubated them for 10 min on a horizontal shaker. Following the staining step aortas were washed with 60% isopropanol, as described above and further rinsed using distilled water. Stained aorta samples were stored in PBS at 4 °C until imaging. Before imaging with Nikon eclipse Ni-U microscope with DS-Ri2 camera (Nikon, Minato, Tokyo, Japan), the stained aortas were cut along their longitudinal axis and opened, making their inner surface visible. Total plaque area in the aortic arch was analyzed using the FIJI® software (National Institutes of Health, Bethesda, MA, USA, https://imagej.net/software/fiji/downloads, accessed on 25 May 2024). The size of the total plaque area (μm^2^) was expressed as a percentage of the total aortic arch.

### 2.6. Statistical Analyses

In the wire myography experiments, Phe-induced contraction data were calculated as normalized to KCl-induced contraction. Statistical analysis was performed with a two-way ANOVA and Holm–Sidak post hoc test for the analyses of comparisons between the eight groups, and a one-way ANOVA with Bonferroni or the Kruskal–Wallis with Dunn tests were used to make comparisons at each concentration level. Values were expressed as the mean ± standard error of the mean (mean ± SEM), and *p* < 0.05 was considered significant. These analyses were performed using the SigmaStat software Version 3.5 (Systat Software Inc., San Jose, CA, USA, www.systat.com, accessed by 1 May 2023) with GraphPad PRISM 9.5.0. (San Diego, CA, USA, www.graphpad.com, accessed by 25 May 2024).

## 3. Results

### 3.1. Plaque Areas Induced by High-Fat Diet

**Figure 1:**
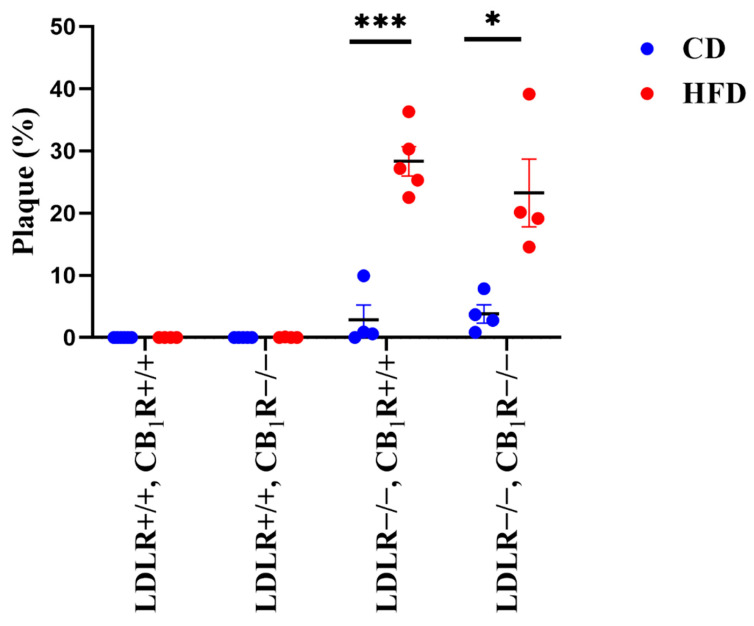
Percentage area of plaques of the aortic arch in mice with genotypes LDLR+/+CB_1_R+/+ (wild-type), LDLR+/+CB_1_R−/−, LDLR−/−CB_1_R+/+ and LDLR−/− CB_1_R−/− kept on control diet or on high-fat diet for 5 months. Mean ±SEM values are shown. (*, *p* = 0.014 and ***, *p* < 0.001 between CD and HFD groups, *n* = 4–6) Abbreviations: LDLR: low-density lipoprotein receptor, CB_1_R: cannabinoid type 1 receptor, CD: control diet, HFD: high fat diet.

**Figure 2:**
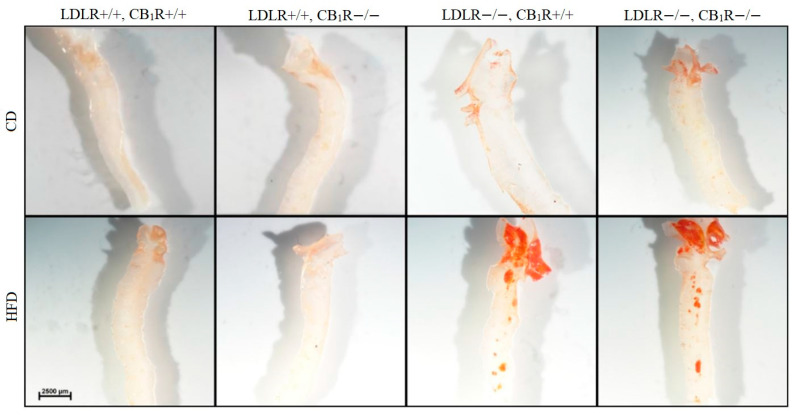
Oil Red stained microscopic image of dissected aortic arch in mice with genotypes LDLR+/+CB_1_R+/+ (wild-type), LDLR+/+CB_1_R−/−, LDLR−/−CB_1_R+/+ and LDLR−/− CB_1_R−/− kept on control diet or on high-fat diet for 5 months. Scale bar indicates 2500 µm. Abbreviations: LDLR: low-density lipoprotein receptor, CB_1_R: cannabinoid type 1 receptor, CD: control diet, HFD: high-fat diet.

Five months of high-fat diet significantly enhanced plaque areas in LDLR-KO animals compared to control diet animals (*p* < 0.05, *n* = 4–6; Figure 1). Also, a rise in plaque areas can be seen in LDLR-KO mice kept on CD compared to LDLR+/+ mice. However, the absence of CB_1_ receptors did not modify plaque density (One-way ANOVA, N.S). Representative photos of Oil Red stained aorta segments from all groups are shown in Figure 2.

### 3.2. Intima–Media Ratio and Wall Thickness of Aortas

**Figure 3:**
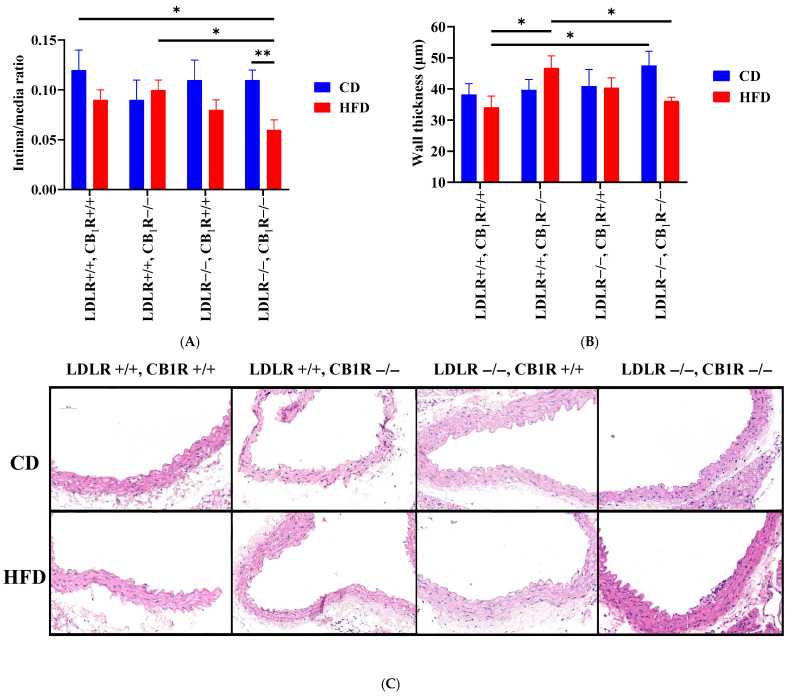
Intima/media ratio (**A**) and wall thickness (**B**) values of hematoxylin–eosin (HE) stained aortas from mice with genotypes LDLR+/+CB_1_R+/+ (wild-type), LDLR+/+CB_1_R−/−, LDLR−/−CB_1_R+/+ and LDLR−/− CB_1_R−/− kept on control diet or on high-fat diet for 5 months. Mean ± SEM values are shown (**A,B**). (*, *p* < 0.05 and **, *p* < 0.01 between groups indicated). (**C**): Representative photos of HE-stained aortas are shown. Abbreviations: LDLR: low-density lipoprotein receptor, CB_1_R: cannabinoid type 1 receptor, CD: control diet, HFD: high-fat diet.

Intima/media ratios were calculated with HE staining. This parameter was higher in CD groups and decreased to HFD in the CB_1_R+/+ groups, whose effect was significant in the LDLR-CB_1_R double-KO group between CD and HFD (from 0.10 ± 0.01 to 0.06 ± 0.01, *p* = 0.007, 1-way ANOVA, Bonferroni test, Figure 3A). Also, intima/media ratios were significant between double-WT-CD and double-KO-HFD (*p* = 0.023, 1-way ANOVA, Bonferroni test) and between double-KO-HFD and LDLR+/+CB_1_R−/−-HFD groups (*p* = 0.028, 1-way ANOVA, Bonferroni test). Wall thickness was significantly higher in LDLR+/+CB_1_R−/−-HFD group compared to double-WT-HFD (*p* = 0.038, 1-way ANOVA, Bonferroni test), and there was a tendency to decrease in double-KO-HFD, compared to CD (NS). Also, there was a significant difference between double-KO-CD and double-WT-HFD groups (*p* = 0.045, 1-way ANOVA, Bonferroni test, Figure 3B).

### 3.3. Elastin Fiber Density Analysis of Tunica Media

**Figure 4:**
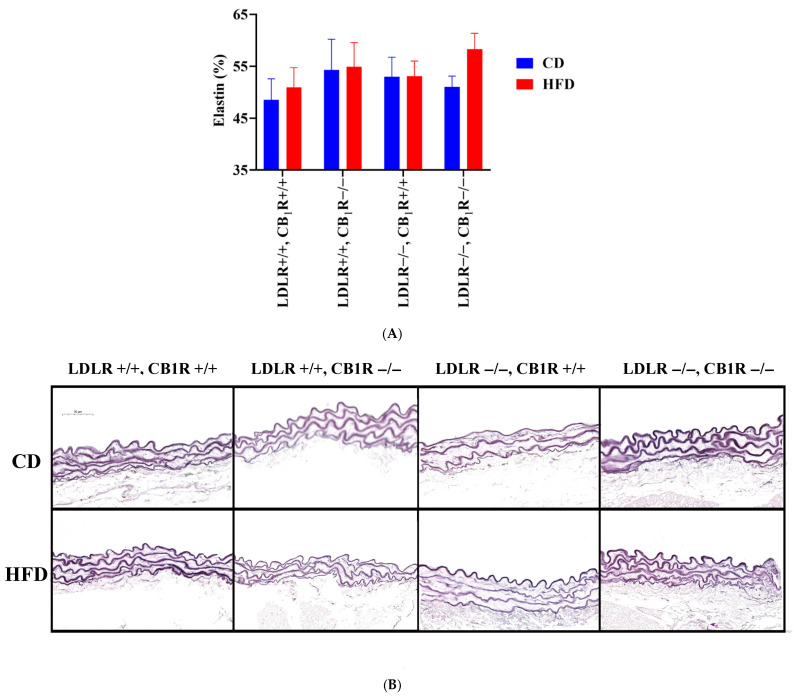
(**A**). Elastin fiber density expressed in elastin % of resorcin-fuchsin stained aortas from mice with genotypes LDLR+/+CB_1_R+/+ (wild-type), LDLR+/+CB_1_R−/−, LDLR−/−CB_1_R+/+ and LDLR−/− CB_1_R−/− kept on a control diet or on a high-fat diet for 5 months. (**B**). Representative photos of RF-stained aortas are shown. Mean ±SEM values are shown (**A**). Abbreviations: LDLR: low-density lipoprotein receptor, CB_1_R: cannabinoid type 1 receptor, CD: control diet, HFD: high-fat diet.

Elastin fiber density expressed as % of medial cross section area was analyzed in RF-stained aortas. Elastin densities were similar in all groups, whose value was the highest in the double KO mice with HFD, (*p* = 0.08 compared to CD group, 1-way ANOVA, pairwise comparisons, Figure 4).

### 3.4. Smooth Muscle Actin

**Figure 5:**
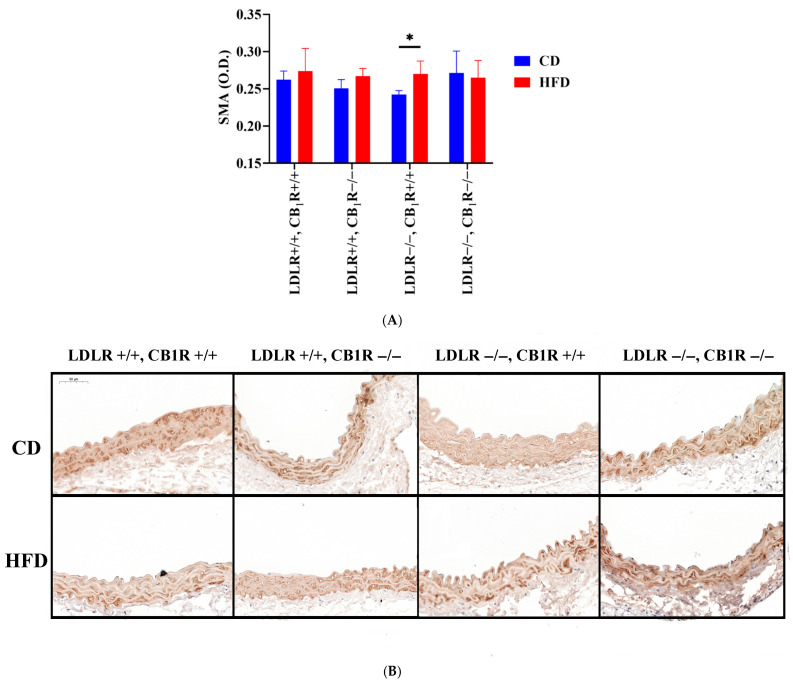
(**A**). Smooth muscle actin immunostaining of aortas from mice with genotypes LDLR+/+CB_1_R+/+ (wild-type), LDLR+/+CB_1_R−/−, LDLR−/−CB_1_R+/+ and LDLR−/− CB_1_R−/− kept on a control diet or on a high-fat diet for 5 months. (*, *p* < 0.05 between groups indicated) (**B**). Representative photos of SMA-stained aortas are shown. Mean ± SEM values of optical density (O.D.) are shown (**A**). Abbreviations: SMA: smooth muscle actin, LDLR: low-density lipoprotein receptor, CB_1_R: cannabinoid type 1 receptor, CD: control diet, HFD: high-fat diet.

SMA immunostaining indicates smooth muscle cell remodeling of the tunica media. SMA level slightly increased in HFD groups whose effect was significant in LDLR−/− CB_1_R+/+ group (*p* < 0.05). This effect disappeared in the double-KO genotype (Figure 5). However, no changes in SMA density in the double KO group may indicate that although HFD initiated a media remodeling, it is absent if CB_1_Rs are missing.

### 3.5. Functional Phenylephrine-Induced Contractile Properties of the Aortas

**Figure 6:**
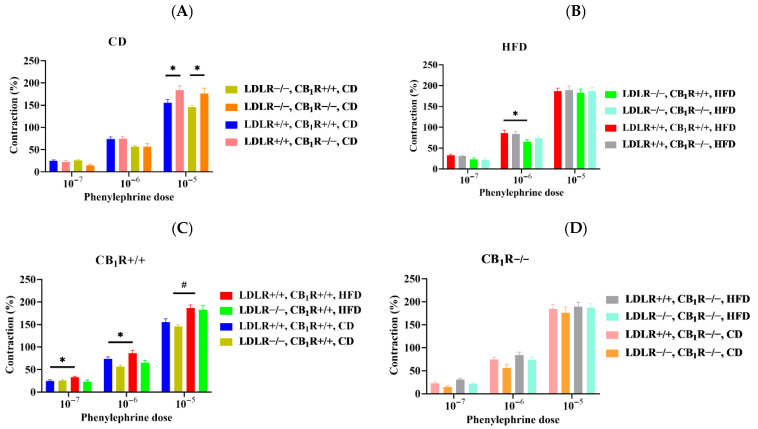
Phenylephrine-induced vasoconstriction of aortas from mice with genotypes LDLR+/+CB_1_R+/+ (wild-type), LDLR+/+CB_1_R−/−, LDLR−/−CB_1_R+/+ and LDLR−/− CB_1_R−/− kept on a control diet or on high-fat diet for 5 months. Panel (**A**): dose–response contraction curves in control-diet groups with different LDLR and CB_1_R genotypes (*n* = 5–7). Panel (**B**): dose–response contraction curves in HFD groups (*n* = 6–10) with different LDLR and CB_1_R genotypes. Panel (**C**): dose–response contraction curves in CB_1_R-wild-type groups with different LDLR genotypes and diets (*n* = 5–10). Panel (**D**): dose–response contraction curves in CB_1_R-knockout groups with different LDLR genotypes and diets (*n* = 6–9). Data are shown as mean ± SEM values. Contraction data are normalized to KCl-induced contraction. Abbreviations: LDLR: low-density lipoprotein receptor, CB_1_R: cannabinoid type 1 receptor, CD: control diet, HFD: high-fat diet. Mean ±SEM values *, *p* < 0.05 between groups indicated and #, *p* < 0.05 between CD and HFD groups.

Phenylephrine-induced vasoconstriction was measured with wire myography. In CD groups, contractions were higher in CB_1_R-KO mice (*p* < 0.05 between LDLR+/+CB_1_R−/− and LDLR+/+CB_1_R+/+ groups such as between LDLR−/−CB_1_R−/− and LDLR−/−CB_1_R+/+ groups (at 10 µM, *p* < 0.05, 1-way ANOVA, Figure 6A). Among HFD groups, there are significant differences between LDLR+/+CB_1_R+/+ and LDLR−/−CB_1_R+/+ groups (at 1 µM, *p* < 0.05, 1-way ANOVA, Figure 6B). Contractions significantly increased to HFD compared to CD groups in CB_1_R+/+ mice (at 10 µM, *p* < 0.05, 1-way ANOVA) and in double WT-HFD compared to CD (at 0.1–1 µM, *p* < 0.05, 1-way ANOVA, Figure 6C), whereas these differences were attenuated in CB_1_R−/− mice (Figure 6D). Further analysis of Phe data and original tracings are shown in Figure S1, Tables S1 and S2 of the Supplementary Materials.

## 4. Discussion

In the present study we investigated the effects of HFD and CB_1_Rs on vascular contractile and structural properties of the aorta from atherosclerotic-prone LDLR-CB_1_R double knockout mouse strain. This mouse strain was established in our laboratory to study the role of ECS and CB_1_ receptors in HFD-induced atherosclerosis. We studied phenylephrine-induced vasoconstriction and structural properties indicated by vascular intima/media ratio, wall thickness, elastin density measured from RF staining and media smooth muscle density applying SMA immunostaining. In the LDLR-KO animal model, HFD effectively increases plasma cholesterol levels [16], and we have also found it in the double LDLR-CB_1_R-KO animals [13].

In order to investigate the effectiveness of our LDLR-CB_1_R double knockout animal model to develop atherosclerosis, after 5 months of HFD we have analyzed plaque areas of thoracic aortas. Our results indicate that long-term HFD-induced plaque formation in LDLR-KO mice groups was not effectively modulated by the absence of the CB_1_Rs.

Related to the functional vascular characteristics of our double KO animal model, our results show that Phe-induced contractions increased to HFD, indicating a functional effect in HFD even in these animals. However, since contractions were even stronger with CD in CB_1_R-KO compared to WT, it may indicate that the presence of CB_1_Rs attenuates initial Phe-induced contractions. This effect conforms with our previous observation that hormone-induced G protein-coupled receptor (GPCR) signaling via calcium-mediated endocannabinoid release can decrease contractile effects induced by vasoconstrictors such as Ang II [24,32,34]. This effect is mediated by 2-AG endocannabinoid, which is produced by the enzyme diacylglycerol lipase (DAGL). In addition, other GPCR-activating hormones are involved in this process. Phenylephrine is known to activate GPCR-mediated calcium signaling via adrenergic α1 receptors, which is also capable to release 2-AG [24,25].

Related to structural vascular parameters, minor structural alterations were observed in animals fed by the HFD, such as decreased intima/media ratio and augmented SMA density (in LDLR-KO-HFD animals compared to LDLR-KO-CD). In the absence of CB_1_Rs, HFD-induced atherosclerotic animals’ plaque area was not modulated; however, the intima/media ratio significantly decreased compared to the CD group and the wall thickness significantly decreased compared to LDLR+/+ HFD animals. Alterations of intima/media ratio are accompanied with limited modulations in wall thickness; these alterations are more prominent in CB_1_R-KO animals. Further, in the absence of the CB_1_Rs, the observed rise in SMA density in HFD disappeared, while elastin density did not show significant changes (with the highest values in double-KO-HFD group).

Despite the fact that knocking out the CB_1_R only moderately modified the vascular contractile and remodeling effects of HFD on atherosclerotic LDLR knockout animals, it positively demonstrates some yet unidentified contribution of the CB_1_Rs to the pathological wall remodeling processes. Our observations demonstrate that HFD-treated LDLR-KO mice develop atherosclerosis at hemodynamically sensitive areas, while in wide areas of the great arteries only functional changes and limited structural alterations can be observed. We have good reason to think that such initial changes may be decisive in more serious pathological processes and should be studied in following investigations.

Our present observations with LDLR-CB_1_R double knockout animals prove that CB_1_Rs contribute to these pathological cell signalization processes.

### 4.1. Vascular Alterations in Atherosclerosis, LDLR-KO Mice

Atherosclerosis is the most common vascular disease manifested in cardiovascular system such as hypertension and stroke [1,2]. Atherosclerosis is a progressive disease usually induced by hypercholesterolemia and mediated by lipid accumulation in the vessel wall. This further develops to vascular inflammation and local plaque formation [1]. Cholesterol with LDL accumulates in the arterial wall subendothelially and transforms into oxidized LDL. This mechanism initiates inflammation by expression of chemotactic proteins, such as monocyte chemoattractant protein-1 and endothelial adhesion molecules promoting the migration of circulating monocytes into the inflamed arterial wall. Monocytes accumulating fat will form foam cells, the essential components of atherosclerotic plaque development [8,10,13,39,40]. The inflammatory response also facilitates the recruitment of circulating monocytes and T cells, which stimulate the migration of vascular smooth muscle cells from the tunica media into the subendothelial space. As a consequence, vascular endothelial dysfunction and unwanted remodeling effects may develop [10,13,14,40,41,42]. As the result of the degenerative vascular remodeling, these events can then turn into plaque formation and necrosis [9,12,42,43,44,45].

In the LDL-receptor-deficient animal model, HFD effectively develops hypercholesterolemia accompanied by atherosclerotic plaques in the aorta, which was shown in several previous studies [12,13,16,18,46]. In this animal model we have also found that 5-month-long HFD induced hypercholesterolemia and atherosclerosis. An important functional alteration was the attenuation of acetylcholine-induced nitric oxide (NO)-dependent vasodilatation in the areas still not affected by the plaques [13]. In the present study we found that the contractile properties of the aorta were increased by HFD, indicating augmented contractile properties with the corresponding vascular damage. However, according to the histology and immunohistology results, only minimal structural vascular changes could be identified, such as a decrease in intima/media ratio and augmented SMA density in the LDLR-KO-HFD group compared to CD.

### 4.2. Vascular Effects of CB_1_ Receptors and Endocannabinoid Signaling, CB_1_R-KO Mice

The ECS contributes to several physiological regulatory mechanisms in the cardiovascular system. Endocannabinoids, such as AEA and 2-AG, as well as synthetic CB_1_R-agonists, such as WIN55,212 can induce acute vasodilatory and hypotensive actions. On the other side, inhibition of the degrading enzyme monoacylglycerol lipase (MAGL) or activating DAG lipase (DAGL) may also augment endocannabinoid levels, further initiating vasodilation or hypotension [6,9,23,24,28,29,30,33,47,48,49]. CB_1_R-dependent vasodilator actions may involve endothelial cells (endothelium-dependent vasodilation), vascular smooth-muscle cells, or perivascular neurons. Such effects are demonstrated in different vascular beds, such as in the aorta, coronaries and cerebral arteries [9,23,24,27,28,29,30,32,48,49]. Endothelium-mediated vasodilation is attributed mostly to nitric oxide (NO), but prostanoids can also be involved [1,32,50,51]. By exogenously administered CB_1_R agonists, endothelium-mediated vasodilatory effects are induced. AEA and 2-arachidonoyl glycerol (2-AG) bind to CB_1_Rs resulting in the hyperpolarization of vascular smooth-muscle cells and causing vasodilation [6,23,24,28,30,33,47,48,49]. CB_1_R-mediated vasodilation can also be elicited by endocannabinoids released during calcium-mediated G_q/11_-protein-coupled receptor activation by contractile agonists. Parallelly, the DAG lipase enzyme will be activated with a consequent release of 2-AG. This way the vasoconstriction effects induced by GPCR agonist hormones can be attenuated by the released 2-AG and coactivation of CB_1_Rs [9,24,25,32,34,35]. A similar mechanism called “retrograde synaptic inhibition” by released endocannabinoids was first discovered in the nervous system [21,52].

Previously we found that WIN55,212-induced vasodilation was absent in CB_1_R-KO mice [24,32,33]. Also, female CB_1_R-KO mice exhibited an augmented NO-dependent relaxation in response to acetylcholine and estradiol [33]. Previously it was proposed that activation of ECS may augment the development of atherosclerosis and plaque formation [53]. Establishing an LDLR-CB_1_R double knockout mouse model provides a tool for studying the involvement of CB_1_Rs in the development of atherosclerosis.

### 4.3. Role of CB_1_ Receptors in Hypercholesterolemia-Induced Vascular Alterations in CB_1_R–LDLR Double-KO Mice

A relationship between the ECS and the development of atherosclerosis was found [54]. It had been described that endocannabinoid signaling could affect atherosclerosis via several mechanisms, influencing vascular inflammation, macrophage mobility and cholesterol metabolism and thus the stability of plaques. In the present study we used a double LDLR-CB_1_R knockout animal model which makes it possible to study the involvement of CB_1_Rs in the development of atherosclerosis. In this model, 5 months of HFD resulted in plaque development in the aorta of LDLR-KO mice, which we have analyzed with Oil Red staining. These mice also have developed extremely high cholesterol levels [13]. In the same model, HFD increased systolic and diastolic blood pressure values in LDLR-KO mice, which was attenuated in the absence of CB_1_Rs. Functional myography studies showed that acetylcholine-induced vasodilation and NO-mediated responses were depressed to HFD compared to CD groups, whereas the absence of CB_1_Rs (in CB_1_R-KO group) could improve the vasodilation and the availability of NO [13].

It was also proposed previously that endocannabinoid signaling is implicated in cardiac dysfunction, inflammation and atherosclerosis, and CB_1_R-antagonism was proposed to be beneficial in these effects [9,55]. Diabetic cardiomyopathy was characterized by an augmented endocannabinoid signaling, activation of mitogen-activated protein kinase (MAPK)-pathway, oxidative-nitrative stress, inflammation and fibrosis, whose effects were attenuated by the inhibition of CB_1_Rs and in CB_1_R-KO mice [9,55].

In the present study we found that the contractile properties of the aorta were increased by HFD. This effect may conform with the previous observation based on the attenuated NO-dependent vasorelaxation effect in HFD [13], which may augment vasoconstrictions. In addition, we have found that in CD animals without atherosclerosis the GPCR agonist Phe-induced vasoconstriction was augmented in the absence of CB_1_Rs. This effect conforms with our previous observations, according to which the agonist-induced vasoconstriction in CB_1_R-KO animals is stronger due to the lack of modulation by signal-induced endocannabinoid release [24,32]. The absence of CB_1_Rs did not change HFD-induced atherosclerotic plaque areas, but it decreased the intima/media ratio compared to the CD group and to LDLR-WT counterparts and also decreased the wall thickness significantly compared to LDLR-WT non-atherosclerotic animals. Alterations of intima/media ratio were accompanied only with minimal alterations of wall thickness values which were more prominent in the absence of CB_1_Rs. The observed rise in SMA density in HFD disappeared, while elastin density was without significant changes in the absence of CB_1_Rs (values were the highest in double-KO-HFD).

Our results indicate that 5 months of HFD induce contractile effects which is not directly modulated by CB_1_R, while some minor structural changes may show an initial arterial wall remodeling. In HFD-induced atherosclerotic animals (LDLR-KO), wall thickness is decreased and the rise in SMA density disappears in the absence of CB_1_Rs. Plaque areas developed in HFD animals were not modulated significantly by the absence of CB_1_Rs. Thus, our data indicate that HFD-treated LDLR-KO mice develop atherosclerosis with functional contractile alterations and moderate structural changes in other arterial areas, and such functional and initial structural alterations are to some degree influenced by CB_1_Rs.

### 4.4. Therapeutic Considerations

Preliminary studies indicate that CB_1_R inhibition might be beneficial in metabolic alterations such as in metabolic syndrome involving obesity or diabetes mellitus as well as in several cardiovascular diseases [9,55,56,57,58]. Exploratory studies on the modulation of CB_1_R or CB_2_R functions might be beneficial in the treatment of cardiovascular diseases such as myocardial infarction, heart failure, cardiac fibrosis and atherosclerosis [9,22,23,26,28,55,56]. In clinical studies, rimonabant and taranabant were previously used for weight reduction in obesity, but unfortunately serious adverse effects appeared [9,57,59].

The selective CB_1_R inhibitor rimonabant was used in atherosclerotic LDL-receptor-knockout mice, which could decrease plaque size in the aorta [60]. Tiyerili et al. found that while CB_1_R inhibition had no effect on the size of atherosclerotic plaques, it has improved endothelium-dependent vasodilation in the aorta [61]. At present, efforts are underway to develop new-generation compounds that can exert therapeutic effects without adverse side effects [9,58,59].

Our present results show that the absence of CB_1_R effect, which can be achieved by chronic pharmacological inhibition, may serve only minimal modulation in the contractile and structural remodeling in the development of atherosclerosis. Issues regarding the beneficial and modulatory effects of CB_1_R inhibition or its absence and the involvement of further downstream endocannabinoid-dependent signaling mechanisms require further confirmation.

### 4.5. Limitations of the Study

There are limitations of the study to be considered. Since eight animal groups were investigated with different genotypes (LDLR- and CB_1_R-KO and -WT animals) subjected to 5 months of a specific diet, the experimental planning depended upon the genotypes of the siblings (for some reason homozygous KO animals had a smaller production rate than WT animals); this process took a reasonably longer time.

Some further limitation is caused by the fact that for plausible reasons, histology, plaque identification and wire myography should be performed on separate segments of the aorta. While plaque development is most prominent in the aortic arch, subjected to hemodynamic forces most intensively, the abdominal aorta yields the most standardized ring preparations for wire myography.

We are convinced that despite the difficulties of establishing the LDLR-CB_1_R double KO mouse strain, such animals provide a useful tool to identify the contribution of the CB_1_R to atherosclerotic wall remodeling processes.

## 5. Conclusions

In the present study, we used the previously established LDLR–CB_1_R double-knockout mouse model, which gives an effective tool to study the involvement of CB_1_Rs in the development of atherosclerosis. In our model, hypercholesterolemia and plaque formation have developed. Our results indicate a modest functional vascular remodeling of the contractile properties to Phe in hypercholesterolemic atherosclerosis-prone LDLR-KO mice fed on an HFD accompanied by only minimal structural alteration in the plaque-free areas. The absence of CB_1_Rs did not effectively modulate plaque formation, but decreased intima/media ratios to HFD with the contractile properties kept higher. Our findings indicate altered functional vascular properties in HFD with increased contraction followed by minimal structural remodeling. The absence of CB_1_Rs was found to elicit higher contraction properties with modest functional differences and with the signs of early vascular remodeling in the atherosclerosis-prone animal model indicating the need for further studies of these pathological cell signalization processes.

## Figures and Tables

**Table 1:** Grouping of animals by genotype and diet.

Group Number	Genotype	Diet	*n*
1.	LDLR+/+; CB_1_R+/+	CD	7
2.	LDLR+/+; CB_1_R−/−	CD	7
3.	LDLR−/−; CB_1_R+/+	CD	8
4.	LDLR−/−; CB_1_R−/−	CD	8
5.	LDLR+/+; CB_1_R+/+	HFD	10
6.	LDLR+/+; CB_1_R−/−	HFD	9
7.	LDLR−/−; CB_1_R+/+	HFD	6
8.	LDLR−/−; CB_1_R−/−	HFD	7

Abbreviations: CD, control diet; HFD, high-fat diet; CB_1_R+/+, cannabinoid type 1 receptor wild type; CB_1_R−/−, cannabinoid type 1 receptor knockout; LDLR+/+, low-density lipoprotein receptor wild type; LDLR−/−, low-density lipoprotein receptor knockout; *n*, number of animals used per group.

## Data Availability

The original contributions presented in this study are included in the article/Supplementary Materials. Further inquiries can be directed to the corresponding author.

## Supplementary Materials

The following supporting information can be downloaded at: https://www.mdpi.com/article/10.3390/biomedicines14020284/s1, Figure S1: Original tracings of myograph recordings of phenylephrine (Phe)-induced vasoconstriction; Table S1: Phenylephrine (Phe)-induced vasoconstriction of aortic rings from mice with genotypes LDLR+/+CB_1_R+/+ (wild-type), LDLR+/+CB_1_R−/−, LDLR−/−CB_1_R+/+ and LDLR−/− CB_1_R−/− (double knockout) kept on control diet or on high-fat diet for 5 months; Table S2: Statistical analysis of phenylephrine (Phe)-induced vasoconstriction of aortic rings of mice with genotypes LDLR+/+CB_1_R+/+ (wild-type), LDLR+/+CB_1_R−/−, LDLR−/−CB_1_R+/+ and LDLR−/− CB_1_R−/−, kept on control or on high-fat diet for 5 months.

## Abbreviations

The following abbreviations are used in this manuscript:

AEA	anandamide
2-AG	2-arachidonoylglycerol
Ang II	angiotensin II
CB_1_	cannabinoid type 1
CB_1_R	cannabinoid type 1 receptor
CD	control diet
DAB	3′3-diaminobenzidine
DAG	diacylglycerol
DAGL	diacylglycerol lipase
ECS	endocannabinoid system
GPCR	G protein-coupled receptor
HE	Hematoxylin–eosin
HFD	high-fat diet
KO	knockout
LDL	low density lipoprotein
LDLR	low density lipoprotein receptor
NO	nitric oxide
O.D.	optical density
PFA	paraformaldehide
RF	resorcin-fuchsin
SMA	smooth muscle actin
VLDL	very-low-density lipoprotein
WT	wild type
WTD	Western-type diet
