# Supplementary material for: Vascular Contractile and Structural Properties in Diet-Induced Atherosclerosis-Prone CB1-LDL Receptor Double Knockout Animal Model
Source: Biomedicines. 2026 Jan 27;14(2):284. doi: 10.3390/biomedicines14020284 (PMC12937606; doi:10.3390/biomedicines14020284)
Supplement: Supplementary file 1 [file biomedicines-14-00284-s001.zip › biomedicines-4082321-supplementary.pdf]

Supplementary material

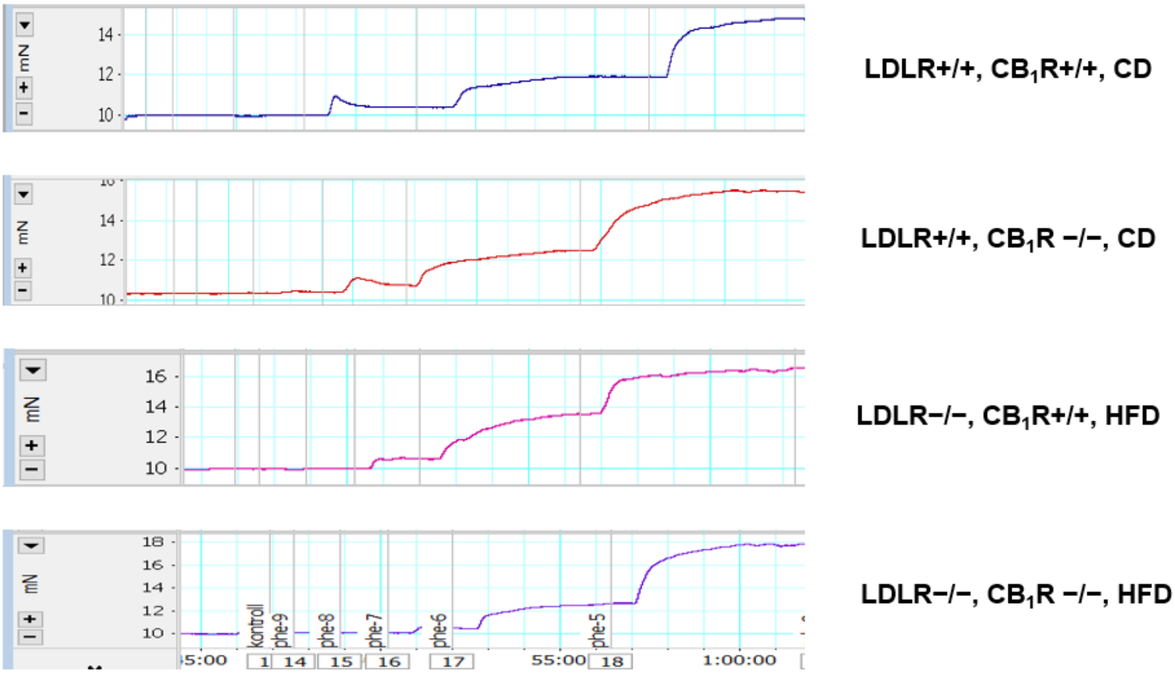

Supplementary Figure S1. Original tracings of myograph recordings of phenylephrine (Phe)-induced vasoconstriction. Data of aortic rings of mice with genotypes and diet groups of LDLR+/+CB1R+/+ (wild-type) CD, LDLR+/+CB1R−/− CD, LDLR−/−CB1R+/+ HFD and LDLR−/−CB1R−/−HFD. Control or high-fat diet for 5 months. Cumulative dose-response curves from 1nM to 10 µM phenylephrine concentrations as indicated. Phe, phenylephrine; LDLR, low-density lipoprotein receptor; CB1R, cannabinoid type 1 receptor; CD, control diet; HFD, high-fat diet.

Supplementary Table S1. Phenylephrine (Phe)-induced vasoconstriction of aortic rings from mice with genotypes LDLR+/+CB1R+/+ (wild-type), LDLR+/+CB1R−/−, LDLR−/−CB1R+/+ and LDLR−/−CB1R−/− (double knockout) kept on control diet or on high-fat diet for 5 months. Contraction maximums in response to 1nM to 10 µM of Phe concentration were normalized to high K+ contractions and expressed as % values. AVG, average (mean) values; SEM, standard error of the mean; Phe, phenylephrine; LDLR, low-density lipoprotein receptor; CB1R, cannabinoid type 1 receptor; CD, control diet; HFD, high-fat diet.

			AVG	AVG	AVG	AVG	AVG	SEM	SEM	SEM	SEM	SEM
Groups			Phe 10-9	Phe 10-8	Phe 10-7	Phe 10-6	Phe 10-5	Phe 10-9	Phe 10-8	Phe 10-7	Phe 10-6	Phe 10-5
1	CD	CB_1R+/+; LDLR+/+	-0,8	3,7	24,8	73,9	155,4	0,2	1,4	2,2	4,6	7,5
2	CD	CB_1R−/−; LDLR+/+	-0,7	1,6	22,8	74,6	184,2	0,3	0,9	2,8	4,9	9,6
3	CD	CB_1R+/+; LDLR−/−	-0,5	2,1	25,7	56,6	145,3	0,2	0,7	2,0	3,5	4,4
4	CD	CB_1R−/−; LDLR−/−	-0,8	-1,0	14,8	56,5	176,4	0,4	0,6	2,7	7,2	12,0
5	HFD	CB_1R+/+; LDLR+/+	-0,1	2,3	33,1	86,4	186,8	0,4	0,7	2,4	6,6	6,8
6	HFD	CB_1R−/−; LDLR+/+	-1,0	1,9	30,9	83,8	189,2	0,3	1,3	2,8	6,7	9,7
7	HFD	CB_1R+/+; LDLR−/−	-1,3	-0,9	23,3	65,0	183,1	0,4	0,6	3,4	5,4	8,8
8	HFD	CB_1R−/−; LDLR−/−	-1,4	-0,8	21,9	73,8	187,1	0,6	0,8	2,4	5,8	8,6

Supplementary Table S2. Statistical analysis of phenylephrine (Phe)-induced vasoconstriction of aortic rings of mice with genotypes LDLR+/+CB_1R+/+ (wild-type), LDLR+/+CB_1R−/−, LDLR−/−CB_1R+/+ and LDLR−/− CB_1R−/−, kept on control or on high-fat diet for 5 months. Contractions at 10 μM of Phe concentration (maximal contractions) are compared. Phe, phenylephrine; LDLR, low-density lipoprotein receptor; CB_1R, cannabinoid type 1 receptor; CD, control diet; HFD, high-fat diet. Analysis was performed with 1-way ANOVA and Bonferroni or Kruskal-Wallis and Dunn tests. P values are indicated.

Groups	1	2	3	4	5	6	7	8
	KD<br>CB1+/+<br>LDL+/+	KD<br>CB1-/-<br>LDL+/+	KD<br>CB1+/+<br>LDL-/-	KD<br>CB1-/-<br>LDL-/-	HFD<br>CB1+/+<br>LDL+/+	HFD<br>CB1-/-<br>LDL+/+	HFD<br>CB1+/+<br>LDL-/-	HFD<br>CB1-/-<br>LDL-/-
1		p=0.027			p=0.005		p=0.023	
2								
3				p=0.03	p=0.001		p=0.001	
4								
5								
6								
7								
8								
